# RNA-Seq explores the functional role of the fibroblast growth factor 10 gene in bovine adipocytes differentiation

**DOI:** 10.5713/ab.23.0185

**Published:** 2023-11-01

**Authors:** Nurgulsim Kaster, Rajwali Khan, Ijaz Ahmad, Kazhgaliyev Nurlybay Zhigerbayevich, Imbay Seisembay, Akhmetbekov Nurbolat, Shaikenova Kymbat Hamitovna, Omarova Karlygash Mirambekovna, Makhanbetova Aizhan Bekbolatovna, Tlegen Garipovich Amangaliyev, Ateikhan Bolatbek, Titanov Zhanat Yeginbaevich, Shakoor Ahmad, Zan Linsen, Begenova Ainagul Baibolsynovna

**Affiliations:** 1 College of Animal Science and Technology, Northwest A&F University, Yangling 712100, China; 2 Faculty of Veterinary and Livestock Technology, S. Seifullin Kazakh Agro Technical University, Astana 010000, Kazakhstan; 3 Department of Livestock Management, Breeding and Genetics, Faculty of Animal Husbandry and Veterinary Sciences, The University of Agriculture Peshawar, 25130, Pakistan; 4 Candidate of Sciences in Agriculture, Researcher of Scientific and Production Centre for Animal Husbandry and Veterinary Limited Liability Partnership, Astana 010000, Kazakhstan; 5 Zhengir Khan West Kazakhstan Agrarian and Technical University, Uralsk 090000, Kazakhstan; 6 Faculty of Agricultural Sciences, Toraighyrov University, Pavlodar 140000, Kazakhstan; 7 College of Veterinary Sciences, Faculty of Animal Husbandry and Veterinary Sciences, The University of Agriculture Peshawar, 25130, Pakistan

**Keywords:** Bovine Adipocytes, *FGF10* Gene, Intramuscular Fat, RNA Sequencing

## Abstract

**Objective:**

The present study was executed to explore the molecular mechanism of fibroblast growth factor 10 (*FGF10*) gene in bovine adipogenesis.

**Methods:**

The bovine *FGF10* gene was overexpressed through Ad-FGF10 or inhibited through siFGF10 and their negative control (NC) in bovine adipocytes, and the multiplicity of infection, transfection efficiency, interference efficiency were evaluated through quantitative real-time polymerase chain reaction, western blotting and fluorescence microscopy. The lipid droplets, triglycerides (TG) content and the expression levels of adipogenic marker genes were measured during preadipocytes differentiation. The differentially expressed genes were explored through deep RNA sequencing.

**Results:**

The highest mRNA level was found in omasum, subcutaneous fat, and intramuscular fat. Moreover, the highest mRNA level was found in adipocytes at day 4 of differentiation. The results of red-oil o staining showed that overexpression (Ad-FGF10) of the *FGF10* gene significantly (p<0.05) reduced the lipid droplets and TG content, and their down-regulation (siFGF10) increased the measurement of lipid droplets and TG in differentiated bovine adipocytes. Furthermore, the overexpression of the *FGF10* gene down regulated the mRNA levels of adipogenic marker genes such as CCAAT enhancer binding protein alpha (*C/EBPα*), fatty acid binding protein (*FABP4*), peroxisome proliferator-activated receptor-γ (*PPARγ*), lipoprotein lipase (*LPL*), and Fas cell surface death receptor (*FAS*), similarly, down-regulation of the *FGF10* gene enriched the mRNA levels of *C/EBPα*, *PPARγ*, *FABP4*, and *LPL* genes (p<0.01). Additionally, the protein levels of PPARγ and FABP4 were reduced (p<0.05) in adipocytes infected with Ad-*FGF10* gene and enriched in adipocytes transfected with siFGF10. Moreover, a total of 1,774 differentially expressed genes (DEGs) including 157 up regulated and 1,617 down regulated genes were explored in adipocytes infected with Ad-FGF10 or Ad-NC through deep RNA-sequencing. The top Kyoto encyclopedia of genes and genomes pathways regulated through DEGs were the PPAR signaling pathway, cell cycle, base excision repair, DNA replication, apoptosis, and regulation of lipolysis in adipocytes.

**Conclusion:**

Therefore, we can conclude that the *FGF10* gene is a negative regulator of bovine adipogenesis and could be used as a candidate gene in marker-assisted selection.

## INTRODUCTION

Qinchuan beef cattle are a dual purpose *Bos taurus* endogenous Chinese breed. It is famous for its quick growth, environmental adaptability, large body frame, and genetic stability. However, its worth is severely reduced, when compared with the marbling characteristics of exotic beef breeds [1–7]. In livestock species, the four adipose tissue sites are intramuscular (IM), intermuscular, visceral, and subcutaneous. The selective enhancement of IM deposition of adipose tissue without affecting other fat depots is a difficult task for the beef industry. Hence, the exploitation of the underlying mechanism of adipogenesis will improve meat quality traits in livestock species [8]. Importantly, the IM fat also known as marbling, is an essential meat sensory characteristic [9,10].

Adipogenesis is the evolution of preadipcytes into mature adipocytes through a multifarious process of terminal differentiation from a multi-potent adipose derived stem cells. The preadipocytes are lifelong present in the body and are capable of specification and differentiation. There are various factors involved in differentiation and maturation of preadipocytes which includes proteins, transcription factors, microRNAs, and epigenetic factors. The exploration of the underlying mechanism of the adipogenesis and lipogenesis is an area of interest for the understanding of the adipocytes role in cardiovascular disease, regenerative medicine, and other obesity related syndromes [11].

Fibroblast growth factors (FGFs) are signaling molecules which perform different vital roles at the cellular level. The family of FGF contains 22 related members with diverse roles in metabolism, neuronal activities, and development. The *FGF10* gene is one of its members which perform a key role in adipose tissue metabolism and development. The *FGF10* gene regulate adipogenesis through CCAAT Enhancer binding protein beta (*CEBPβ*) via autocrine/paracrine mechanism [12]. The *FGF10* gene mediates proliferation of preadipocyte through MAPK pathway, and phosphorylation of *p130* gene through cyclin D2 dependent Ras- MAPK pathway. Furthermore, the *FGF10* gene induces the transcription of retinoblastoma protein (*pRb*) gene which binds with *CEBPα* and thus stimulates adipogenesis through Ras-MAPK pathway [13]. Previously, the *FGF10* gene expression was down-regulated in adipocytes which inhibited the expression of CEBPβ and subsequently differentiation of adipocytes [12]. Adipogenesis, being a complex biochemical process, involves differentiation of preadipocytes whereas proliferation and maturation of adipocytes. Preadipocytes, which originate from the existent group of adipocytes undergoes the process of development in response to suitable stimuli [14]. Therefore, it is essential to better understand the molecular basis of adipogenesis. Previously, we explored the association of genetic polymorphism of *FGF10* gene promoter with meat quality characteristics in Qinchuan beef cattle [15]. However, the in-depth molecular mechanism of *FGF10* gene in bovine adipocytes still needs to be explored. Hence, the present study was executed to exploit the functional role of *FGF10* gene in bovine adipogenesis.

## MATERIALS AND METHODS

### Ethical statement

All animal experiments took place at “National Beef Cattle Improvement Research Center, Northwest A&F University, Yangling, China. The procedures regarding animal handling were carried our as per guidance and approval of the animal care and ethical committee of the Northwest Agriculture and Forestry University, Yangling China vide notification No.NWAFU/AST/69.

### Collection and preservation of test samples

The tissue samples of healthy newborn Qinchuan beef cattle were collected from the National Beef Cattle Improvement Research Center (NBCC) of the Northwest A&F University, China. After animals were humanly euthanized, the samples were aseptically collected from thirteen tissues including omasum, subcutaneous fat, IM fat, lung, rumen, abomasum, reticulum, spleen, small intestine, kidney, heart, liver, and muscle as described previously [16,17]. The samples were cryopreserved at −80°C in refrigerator for subsequent experiments.

### Isolation of preadipocyte cells

The adipose tissue was aseptically collected from the longissimus dorsi muscle area. The tissue was first washed with 10% penicillin and streptomycin-phosphate-buffered saline (PBS) (Invitrogen, Carlsbad, CA, USA) solution. In the cell culture room, the adipose tissue was detached from the blood vessels and connective tissues with the help of sterile forceps under stereo dissecting microscope. The adipose tissues was digested with collagenase I enzyme-0.25% (Sigma, Shanghai, China) at 37°C for I hour, and then neutralized with 10% fetal bovine serum (FBS). The mixture was first filtered through 100 μm and then with 40 μm strainers, centrifuged for 10 minutes at 1,500×g. The filtrate was pelleted and washed with medium DMEM/F12 containing 10% FBS (Gibco, Grand Island, NY, USA), seeded in collagen coated 60 mm plates, and incubated for one hour at 37°C in 5% CO_2_.The medium was aspirated, cells were washed with PBS to remove the dead cells, and fresh medium was added to the cells.

### Vector construction and determination of the best multiplicity of infection

**Figure 1:**
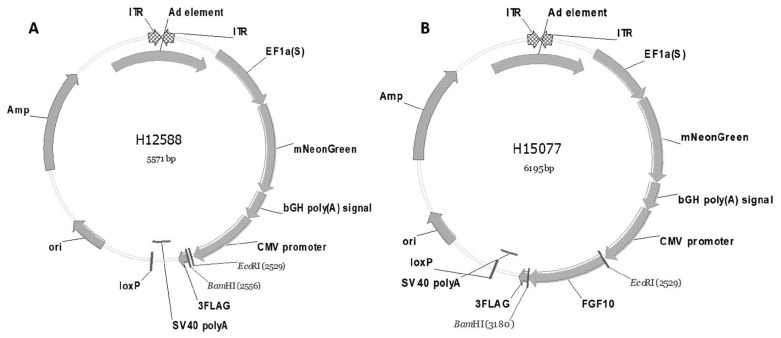
Adino-virus packaging of *FGF10* gene. (A) The adenovirus vector represent the insertion site of the *FGF10* gene. (B) The adenovirus vector with the inserted *FGF10* gene. *FGF10*, fibroblast growth factor 10.

The pAdeno-EF1A(S)-mNeonGreen-CMV-FGF10-3FLAG adenovirus vector were synthesized through Shanghai Heyuan Biotechnology Co., Ltd. and was used for the overexpression of *FGF10* gene (Figure 1). The optimal multiplicity of infection (MOI) value and overexpression efficacy of the virus were determined. The isolated primary preadipocytes were inoculated in a 12-well plate, and the MOI value was calculated according to the virus titer and cell number. When the cell number reached 80% to 90% confluence, the preadipocytes were infected with Ad-FGF10 and negative control (NC) at different gradient MOI values (5, 10, 25, 35, 50, 65), the cell morphology was observed after 48h post infection, and the most suitable MOI value was determined based green fluorescence and cell morphology characteristics.

### Overexpression efficiency assay

The bovine preadipocytes were passage into the 6-well plate and infected drop wise with Ad-FGF10 and Ad-CMV-NC viruses at 80% to 90% confluence of the cells according to the optimal MOI value. After 24 hours after infection, the culture medium was changed, then 48 hours post infection, the cells were collected to extract the total RNA and subjected to reverse transcription for construction of cDNA. The relative mRNA level of the *FGF10* gene was quantified in both overexpression and control groups at both mRNA and protein levels. The list of the primers is available in Supplementary Table S1.

### Determination of siRNA transfection and interference efficiency determination

The detection of transfection efficiency was performed according to the fluorescently labeled siRNA (FAM-siRNA) according to the instructions of lipofectamin 3,000 transfection reagent. Bovine preadipocytes were cultured in 6 well plates, and when they grew to 70% to 90% confluence, cells were starved for 2 h with serum-free medium, and 3.75 μL lipofectamin 3000 (Invitrogen, USA). The FAM-siRNA oligos and transfection reagent were mixed in medium (Opti-MEM; Gibco, USA) separately, the mixtures were left stand for 10 minutes at room temperature. Both mixtures were combined, mixed through vortex, and again left stand for 15 minutes at RT (room temperature). The mixture of FAM-siRNA and transfection reagent poured drop wise into the cells and incubated for 24 hours in 5% CO_2_ at 37°C. The cell morphology and fluorescence were checked under a fluorescence microscope in a dark room at 24 hours of post infection.

### Induced differentiation of bovine preadipocytes and red oil o staining

The adipocytes were infected drop wise with Ad-FGF10 or siFGF10 and their NCs at 80% to 90% confluence of the cells according to the optimal MOI value, at density of 1.2×105 cells with transfection reagent lipofectamine Lipo-3000 (Invitrogen, USA). The cells were induced differentiated with first differentiation media including 1 μM dexamethasone, 0.5 mM hydro cortisol, 0. 5 mmol/L of 3. isobut-1-methylxanthine (IBMX), and 167 nM insulin at 24 hours of post infection [18]. At 2 days post-infection, the culture medium was switched to the second differentiation medium including DMEM/F-12, 10% FBS, and 5 μg/mL insulin. The adipocytes were first washed 2 to 3 times with PBS, and then 4% of paraformaldehyde was added for 30 min for fixation of the cells. First, 1 mL oil red O staining solution was added dip wise to the culture plate and incubated 30 minutes under dark at room temperature. The oil red O staining solution was then aspirated from the culture plate which was washed 2 to 3 times with PBS and the cells observed under an inverted phase-contrast microscope. The photographs were captured, and the lipid droplets were measured with ImageJ software.

### Extraction of total RNA, construction of cDNA, and qRT-PCR

The total RNA was extracted from the adipocytes through TRIZOL method (Takara, Beijing, China). The concentration and quality of the extracted RNA were assessed through optical density (OD) of the 260, its ratio 260/280 using Nanodrop TM (TECAN), and 1% agarose gel. The RNA was reverse transcribed into cDNA through Prime-Script RT reagent kit (Takara, China). The quantitative polymerase chain reaction (qPCR) was performed through SYBR- Premix ExTaq II kit (Takara, China). The *β actin* and *GAPDH* were used as house-keeping genes and the relative mRNA level of the target genes were measured through 2^−ΔΔCt^ method.

### Preparation of total RNA for sequencing

The preadipocytes were cultured in 6-well plate and infected with Ad-FGF10 and Ad-NC at 70% to 80% confluence of the cells. On day 04 of induced differentiation, the total RNA was collected by Trizol method (Invitrogen, Carlsbad, CA, USA), and its quality was analyzed with Bioanalyzer, Agilent-2100 (Agilent Technologies, Palo-Alto, CA, USA) and with gel electrophoresis. The Oligo-dT beads were mixed with total RNA to enrich the mRNA and short fragments were made with fragmentation buffer, and finally a cDNA library was constructed with random primers. The second strand cDNA was generated with polymerase-I, RNase H, buffers, and dNTPs. The fragments were purified with Qia-Quick PCR extraction kit (Qiagen, Shanghai, China), and the poly-A was paired-end into Illumina sequencing adapters. The end products were gel purified from the agarose gel through electrophoresis, then amplified with PCR, and sequenced through Illumina2500 via Gene Denovo Biotechnology Co. (Guangzhou, China).

### Bioinformatics analysis

#### Clean reads filtration

The raw data of the sequencing reads including low quality base pairs and adapters (>10% of the unknown base pairs, and more than fifty percent low quality base pairs with q-value of more than 20) were excluded using a computer software fastp-v 0.18 [19].

#### Alignment of reads with rRNA (Ribosome RNA)

The alignment tool Bowtie-2 software were used for mapping the reads with rRNA [20]. The clean reads of the data were aligned with reference genome through “rna-strandness RF” through HISAT2. 2.4 software [21].

#### Gene abundance quantification

The fragment per kilobase of transcript per million mapped reads (FPKM) were measured through String-Tie software [22] using following formula.


$$\text{FPKM}=\frac{10^{6}\text{C}}{\text{NL}/10^{3}}$$

Where FPKM (A) represent expression of gene A; C shows fragment numbers aligned to gene A; the total numbers of fragments aligned with the reference genome were shown with N; and L shows the number of base pairs in gene A.

#### Identification of deferential expressed genes

The deferential expressed genes (DEGs) were explored through DE-Seq-2 software [23]. The variation between the two groups were measured through edge-R software [24]. The significant DEGs were screened with fix parameters as criteria false discovery rate (FDR) <0.05, |log2FC|>1, and absolute fold change ≥2. The expression and clustering of DEGs in each sample were presented through a heat map.

#### Gene ontology and Kyoto encyclopedia of genes and genomes enrichment analysis

In this study, the DEGs were aligned to each term of the gene ontology (GO) database (http://www.geneontology.org/). The number of differential genes in each term was calculated, and the list and numbers of differential genes under the function of each GO entry were counted. Items with corrected p-value as q-value less than 0.05 were considered significantly enriched items using the following formula. The DEGs were aligned to each pathway in the Kyoto encyclopedia of genes and genomes (KEGG) database (https://www.kegg.jp/). Hyper geometric test explored significantly enriched pathways in differential genes based on the reference genome. The pathway with corrected p-value as q-value less than 0.05 were considered significantly enriched using the following formula.


$$P=1-\sum_{i=0}^{m-1}\frac{\left(\begin{array}{c} M\\ i \end{array}\right)\left(\begin{array}{c} N-M\\ n-i \end{array}\right)}{\left(\begin{array}{c} N\\ n \end{array}\right)}$$

Where N represents the number of all transcripts in GO annotation, the n shows numbers of DEGs in N, M depicts the numbers of transcripts GO terms, m shows the numbers of DEGs in M. The FDR correction (≤0.05) was considered as significantly enriched GO terms in DEGs. The same formula was used for KEGG pathways enrichment analysis.

### Statistical analysis

The computer software SAS.8 (SAS Institute, Cary, NC, USA) was used for the analysis of variance, and Graph-Pad Prism-6 (GraphPad, San Diego, CA, USA) were used for the graph designing and statistical variation. The “p<0.05” were considered as statistically significant.

## RESULTS

### Differentiation efficiency of preadipocytes and expression pattern of *FGF10* gene

**Figure 2:**
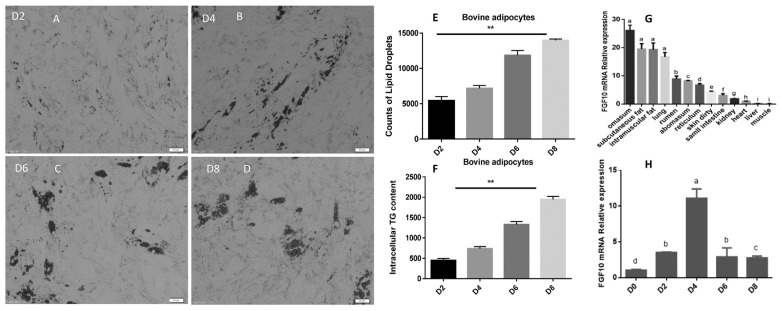
Differentiation efficiency of bovine preadipocytes and expression of FGF10 in adipogenesis. (A–F) Lipid droplets and TG contents during induced differentiation (day 2 to day 8) of bovine adipocytes. (G) Expression level of FGF10 in different tissues (H) Expression level of *FGF10* gene in different days of bovine adipocytes differentiation. FGF10, fibroblast growth factor 10; TG, triglyceride. ^a–i^ p<0.05, ** p<0.01.

First, we validated the differentiation efficiency of the adipocyte cells. The lipid droplets counts were significantly (p< 0.05) increased in cells during differentiation from day 1 to day 8 (Figure 2A–2E). Moreover, a similar trend was observed in the triglycerides (TG) content of adipocytes during bovine adipocyte differentiation (1F). To illuminate the function of bovine *FGF10* gene in adipogenesis, we examined the expression pattern of *FGF10* gene through qRT-PCR analysis. The mRNA expression of *FGF10* gene in omasum, subcutaneous fat and IM fat was significantly enriched compared with other tissues (p<0.05) as shown in figure IG. Furthermore, significantly (p<0.05) highest expression of *FGF10* gene was found in day 4 of induced differentiation in bovine preadipocytes, however the expression level declined from mid to late differentiation on day 8 (Figure 2H). These results revealed that *FGF10* gene possibly plays a vital role in adipogenesis.

### Determination of optimal multiplicity of infection of Ad-CMV-FGF10 virus, and overexpression and inhibition efficiencies

The cells were cultured in 12-wells plates and infected with either Ad-FGF10 or Ad-CMV-NC in different proportions at MOI values of 5, 10, 25, 35, 50, 65 at 80% to 90% confluence of the cells. The MOI value was calculated according to the virus titer and cell numbers. After 48 hours of infection, the cell morphology was observed under fluorescence microscope. Interesting, the MOI values of Ad-FGF10 and Ad-CMV-NC were 25 and 5 respectively, the cells had a good growth state, and no obvious lesions were found (Figure 2A–2D). At the same time, after 48h, the total RNA and total protein of the cells were collected, and the overexpression efficiency of FGF10 was detected at the mRNA and protein levels, respectively. Compared with the control group, the expression level of *FGF10* gene mRNA increased by more than 2,800 times (Figure 2E); and the FGF10 protein expression level increased by 17 times compared with the NC group (Figure 2F). The results indicated that the adenovirus successfully mediated the overexpression of *FGF10* gene in bovine preadipocytes, effectively improved the overexpression efficiency of FGF10, and could be used in downstream experiments. The interference efficiencies of the three siRNAs including si-622, si-348, si-142 and siNC (Figure 2G; Supplementary Table S1) were evaluated and the best one was selected for downstream application (2H). Briefly, after 48 hours of adipocytes transfection, the total RNA were extracted from the cells and cDNA library were constructed. The qRT-PCR was performed for the detection of the interference efficiency of the selected siRNA, using the β-actin as an internal reference gene. The qRT-PCR analysis shows that, si-622 interferes 40% expression of *FGF10* gene, si-348 67%, and si-142 inhibited 55% mRNA expression of *FGF10* gene. Therefore, si-348 exhibited highest (p<0.01) interference efficiency against *FGF10* gene at mRNA level. Interestingly, the WB results shows similar results and si-348 down-regulated the FGF10 expression by 60% at protein level (Figure 2H). Moreover, the cell morphology was also not affected, therefore, si-348 was selected and used in downstream experiment for the inhibition of *FGF10* gene in bovine adipocytes.

### Role of *FGF10* gene in differentiation of adipocyte

**Figure 3:**
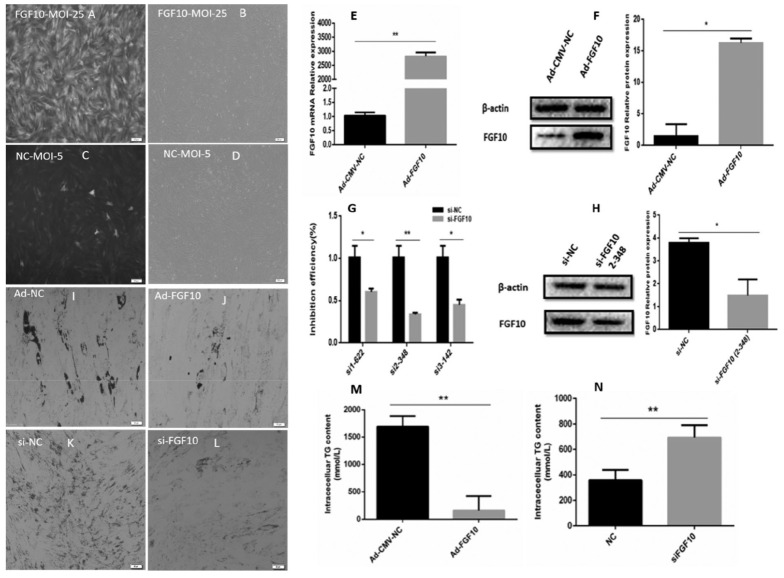
Optimization of MOI value and over-expression and inhibition efficiencies of adiono-FGF10 and si-FGF10 in bovine adipocytes. (A–D) Determination of optimum MOI value in bovine adipocytes (E) The mRNA expression of *FGF10* gene in adipocytes infected with Ad-FGF10 and Ad-NC for the evaluation of overexpression efficacy. (F) The protein expression of *FGF10* gene in adipocytes infected with Ad-FGF10 and Ad-NC for the evaluation of overexpression efficacy. (G) The mRNA expression of *FGF10* gene in adipocytes transfected with three siRNAs (si-622, si-348, si-142 against their NC) for the analysis of Interference of *FGF10* gene. (H) Protein expression of *FGF10* gene in adipocytes transfected with si-348and NC to validate the Interference of *FGF10* gene. (I–J) The lipid droplets in bovine adipocytes transfected with Ad-FGF10 and Ad-NC. (K–L) The lipid droplets in adipocytes transfected with si-348 and NC. (M) The triglyceride content in adipocytes infected with Ad-FGF10 and Ad-NC. (N) The triglyceride content in bovine adipocytes infected with si-348 and NC. MOI, multiplicity of infection; FGF10, fibroblast growth factor 10; NC, negative control. * p<0.05, ** p<0.01.

To further illuminate the role of *FGF10* gene in adipogenesis, the lipid droplets were quantified in adipocytes infected with Ad-FGF10 and Ad-NC. The red-oil o staining results showed that, overexpression of *FGF10* gene significantly (p<0.05) reduced the lipid droplets measurement in differentiated bovine adipocytes (Figure 3I–3J). Similarly, down-regulation of *FGF10* gene increased (p<0.05) the measurement of lipid droplets in differentiated bovine adipocytes (Figure 3K–3L). Additionally, the TG content was also reduced in adipocytes infected with Ad-*FGF10* gene (Figure 3M), and the content of TG increased in the adipocytes transfected with si*FGF10* gene as compared with siNC (Figure 3N). These results shows that FGF10 negatively regulates bovine adipogenesis.

**Figure 4:**
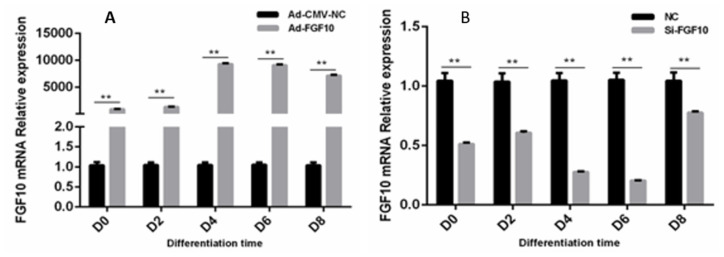
The relative mRNA level of *FGF10* gene in adipocytes. (A) The mRNA level of *FGF10* gene infected with Ad-FGF10 during the course of differentiation at D0, D4, D6, and D8. (B) The mRNA level of *FGF10* gene transfected with siFGF10 during the course of differentiation at D0, D4, D6, and D8. FGF10, fibroblast growth factor 10. ** p<0.01.

To further explore the role of *FGF10* gene in adipocyte differentiation, the *FGF10* gene was over-expressed or inhibited during different stages of induced differentiation at 0, 2, 4, 6, and 8 d (Figure 4A–4B). The highest mRNA expression level was found in adipocytes infected with Ad-*FGF10* gene at day-4 of differentiation. Similarly, the highest interference efficiency was found in day-6 of induced differentiation. These findings suggests the role of *FGF10* gene in the middle stage of adipogenesis. Therefore, we selected adipocytes infected with Ad-*FGF10* gene and Ad-NC at day-4 of differentiation for deep RNA sequencing analysis.

**Figure 5:**
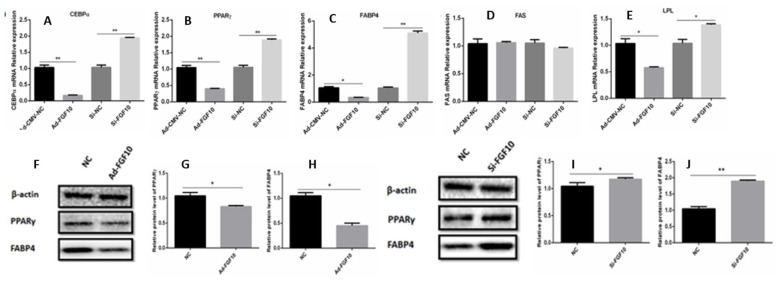
The mRNA and protein level expression of the adipognesis related genes in adipocytes transfected with Ad-FGF10, si-FGF10 and their respective NCs. (A–E) the mRNA expression level of *CEBPα*, *PPARγ*, *FABP4*, *FAS* and *LPL* genes in adipocytes transfected with Ad-FGF10, si-FGF10 and their respective NCs. (F–J) The relative protein level of PPARγ and FABP4 in adipocytes transfected with Ad-FGF10, si-FGF10 and their respective NCs. FGF10, fibroblast growth factor 10; NC, negative control; *CEBPα*, CCAAT enhancer binding protein beta; *PPARγ*, peroxisome proliferator-activated receptor-γ; *FABP4*, fatty acid binding protein 4; *FAS*, Fas cell surface death receptor; *LPL*, lipoprotein lipase. * p<0.05, ** p<0.01.

To further explicate the role of *FGF10* gene in the adipogenesis, we quantified the mRNA and protein of adipogenesis marker genes in differentiated adipocytes. Enrichment of *FGF10* genes in bovine adipocytes at day 4 of differentiation, the expression level of peroxisome proliferator-activated receptor-γ (*PPAR*γ), CCAAT enhancer binding protein alpha (*CEBPα*), Fas cell surface death receptor (*FAS*), lipoprotein lipase (*LPL*), and fatty acid binding protein (*FABP4*) genes were down-regulated (p<0.01) as shown in Figure 5A–5E. Similarly, down-regulation of the *FGF10* gene enriches the mRNA levels of *PPAR*γ, *CEBPα*, *LPL*, and *FABP4* genes (p<0.01) at day 6 of adipocyte differentiation (Figure 5A–5C, 5E). Moreover, the protein expression of PPARγ and FABP4 were reduced (p<0.05) in adipocytes infected with Ad-*FGF10* gene at day 4 of differentiation (Figure 4F–4H). Strikingly, the protein expression of PPARγ and FABP4 were enriched (p<0.05) in adipocytes transfected with si*FGF10* gene at day 6 of differentiation (Figure 5I–5K). These findings further predicts the role of *FGF10* gene in adipocytes differentiation.

### Quality control of the data used for RNA sequencing

**Figure 6:**
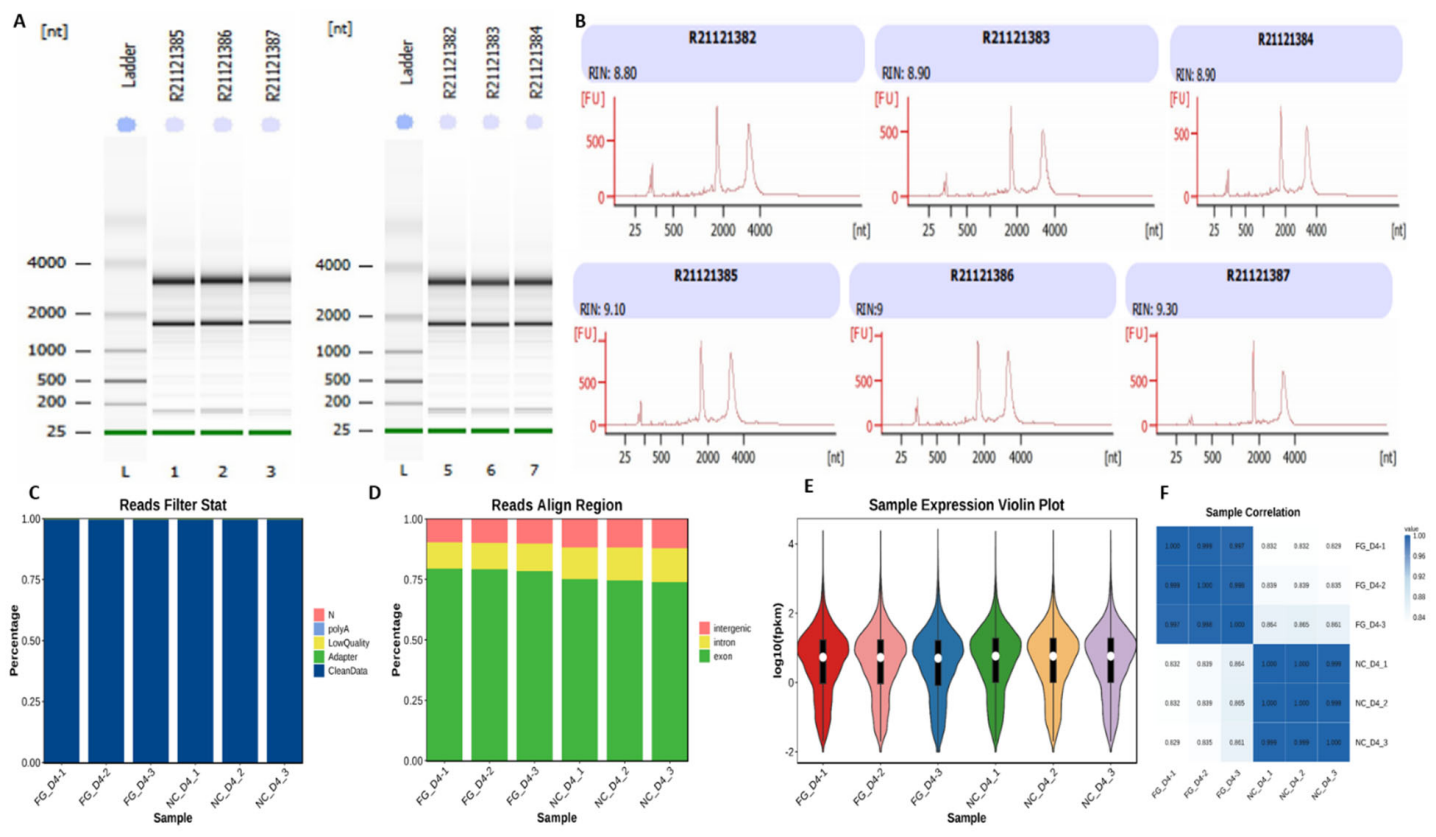
RNA samples quality and data analysis. (A–B) Gel electrophoresis and RIN values of the RNA samples extracted from adipocytes infected with Ad-FGF10 and NC. (C–D) The sequenced data validity extracted from the adipocytes infected with Ad-FGF10 and NC. (C) The clean read filtration of the data shown with blue after removal of the low quality base pairs and adapters. (D) The aligned clean reads were shown with various colors and represents various regions of the genome. Red color shows inter-genic region; yellow color shows intronic region; and green color shows exonic region. (E) The FPKM in each sample of adipocytes infected with Ad-FGF10 and NC. (F) The heat map correlation of the two group’s adipocytes samples transfected with adino-FGF10 and NC. FPKM, fragments per kilobase million; FGF10, fibroblast growth factor 10; NC, negative control.

Three biological replicates were made in each category of the differentiated adipocytes infected with Ad-FGF10 (n = 3) and the Ad-NC (n = 3), and a total of 6 cDNA libraries were constructed. The RNA quality was evaluated as represented with gel electrophoresis of the RNA samples and RIN (RNA integrity number), which ranged from 8.50 to 10 (Figure 6A–6B). Moreover, the clean reads were aligned to the reference genome of *Bos taurus* (Figure 6C–6D). The unique mapped sequence (Unique Mapped) of each sample is over 90%, and the total alignment rate is over 92%. It occupies a major share in the alignment rate and fulfill the requirements of the data volume for RNA sequence analysis (Table 1). Based on the alignment results of the reads mapped to the genome, the distribution positions were calculated in the reference genome, and most of the sequencing reads were compared to the exon region, and the matching rate was over 75% (6C–6D).

Additionally, the significance of the transcriptomic analysis was also confirmed through expression abundance of the samples (Figure 6E). The correlation between the samples of the two experimental groups was more than 80%, while the correlation between the samples within the group was over 99%. These findings shows reliability of the data and hence validate the use of samples for downstream application (Figure 6F).

**Figure 7:**
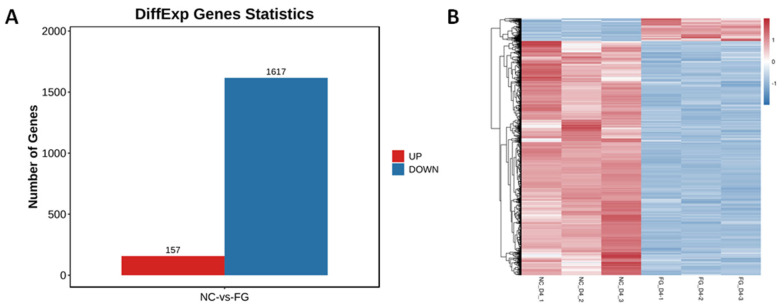
Quantification of DEGs in adipocytes infected with Ad-FGF10 and Ad-NC. (A) The number of differential gene expression exhibited by the bar graph within two categories of adipocytes infected with Ad-FGF10 and Ad-NC (B) The hierarchical cluster analysis represented with heat map of DEGs in two categories of adipocytes infected with Ad-FGF10 and Ad-NC (B). Blue color depicts down-regulated and red color shows up-regulated genes. DEGs, differentially expressed genes; FGF10, fibroblast growth factor 10; NC, negative control.

Through differential analysis, a total of 1,774 significantly different genes were obtained, including 157 genes up regulated and 1,617 genes down regulated (Figure 7A). The clustering of expression levels of differential genes among samples is shown in Figure 7B. The results show that the expression levels of the enriched genes and down regulated genes are different (p<0.05) in adipocytes infected with Ad-FGF10 (n = 3) and the Ad-NC (n = 3).

**Figure 8:**
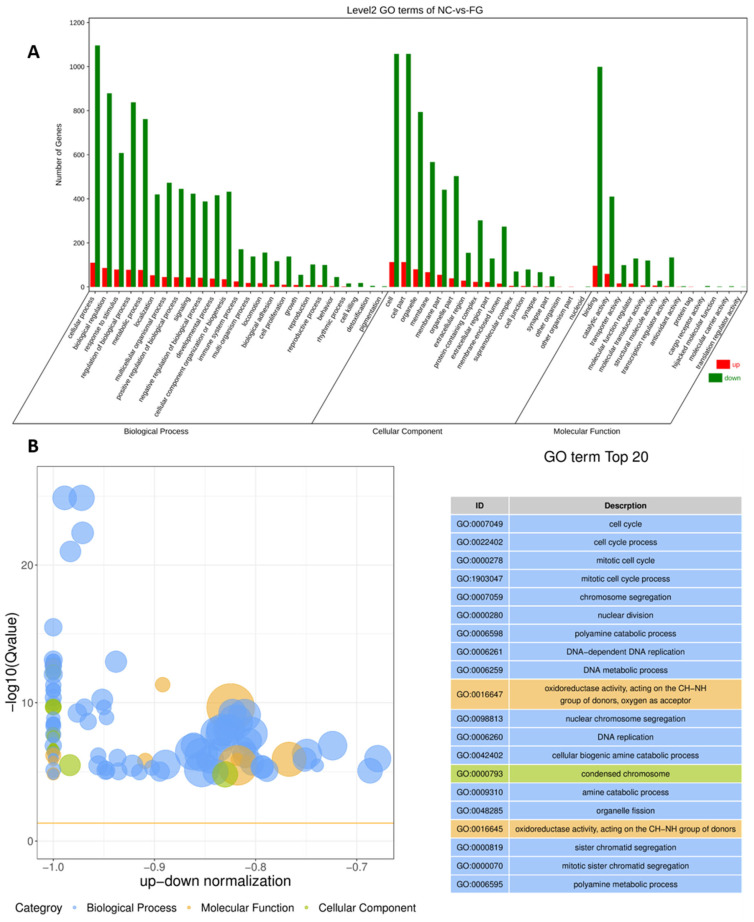
The GO classification of DEGs in two categories of adipocytes infected with Ad-FGF10 and Ad-NC (A) The GO terms of the unigenes explored through transcriptomic analysis in two categories of bovine adipocytes infected with Ad-FGF10 and Ad-NC. (B) The Z score in GO category are shown with BP (blue), CC (green), and MF (yellow). The top twenty GO terms enriched due to DEGs in two categories of adipocytes infected with Ad-FGF10 and Ad-NC. GO, gene ontology; DEGs, differentially expressed genes; FGF10, fibroblast growth factor 10; NC, negative control; BP, biological process; CC, cellular component; MF, molecular function.

The up regulation of *FGF10* significantly (p<0.05) mediated the GO terms (Figure 8A). The overexpression of the *FGF10* gene revealed three functional levels of GO terms comprising cellular component (CC), molecular function (MF), and biological process (BP). The BP was the highest contributor (80.08%) to the GO enrichment terms, followed by 13.4% (CC) and 6.7% (MF). The top twenty GO terms significantly enriched due to DEGs in bovine adipocytes are shown in figure 8B. These GO terms including cells cycle process, chromosomal segregation, mitotic cells cycle, nuclear division, DNA replication, and DNA metabolics process.

**Figure 9:**
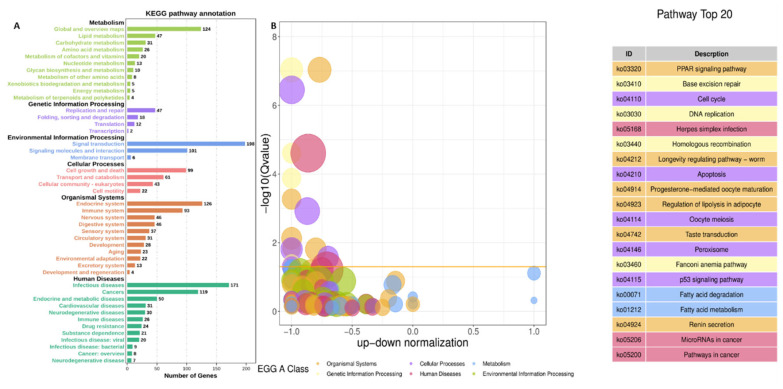
The KEGG pathways regulated by DEGs in two categories of adipocytes infected with Ad-FGF10 and Ad-NC. (A) Top twenty KEGG pathways enriched by DEGs in in two categories of adipocytes infected with Ad-FGF10 and Ad-NC. (B) The bubble chart of the KO enrichment analysis, the yellow color shows Q-value as <0.05 a threshold value. KEGG, Kyoto encyclopedia of genes and genomes; DEGs, differentially expressed genes; FGF10, fibroblast growth factor 10; NC, negative control.

The top twenty significantly (Q<0.05) enriched KEGG pathways validated the roles of DEGs modulated by the *FGF10* gene in adipocytes (Figure 9). The KEGG A class enriched pathways are metabolisms (lipid, carbohydrates, amino acid, cofactor and vitamins, and nucleotide metabolisms) genetic information processes (transcription, translation, replication, and repair); environmental information process (signaling molecule and interactions, and signals transduction); cellular process (transport and catabolism, cellular growth and death, cell motility, and cellular community eukaryote); and organism systems (endocrine system, immune system, sensory system, nervous system, digestive system, circulatory system, environmental adaptation, development, and aging); diseases (infectious diseases, tumors, metabolic and endocrine diseases, cardiovascular and neurodegenerative diseases). These pathways of the KEGG A class play significant roles in adipogenesis. The top twenty pathways regulated with enrichment of *FGF10* gene in bovine adipocytes includes PPAR signaling pathway, bases excision repairs, cells cycle, DNA replications, herpes simplex infections, homologous recombination, apoptosis, and regulation of lipolysis in adipocytes (Figure 9B). Furthermore, to explore the effect of *FGF10* gene in the differential transcriptomics pattern of FGF receptors in differentiated bovine adipocytes infected with Ad-FGF10 or Ad-NC, we extracted the transcriptomics profile of the selected FGF receptors from the RNA sequencing data (Table 2). The results showed that enrichment of *FGF10* gene in differentiated bovine adipocytes down-regulated the expression of *FGFRL1*, *FGFR2*, *FGFR4*, and *FGFR3*.

### Validation of RNA sequencing results

**Figure 10:**
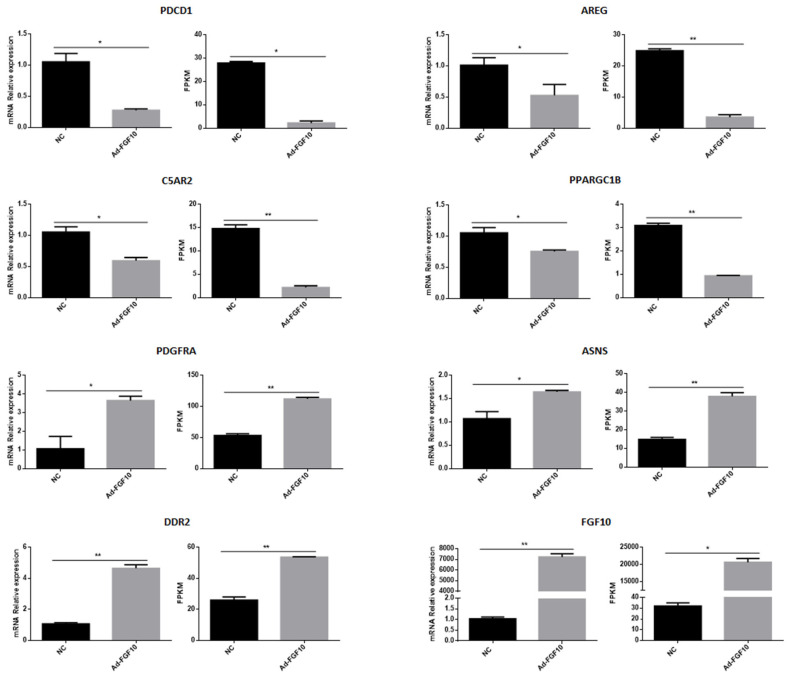
The expression of selected gene analyzed through quantitative real-time polymerase chain reaction analysis. * p<0.05, ** p<0.01.

To validate the sequencing results, eight DEGs including programmed cell death protein (*PDCD1*), amphiregulin (*AREG*), C5a anaphylatoxin chemotactic receptor 2 (*C5AR2*), PPARG Coactivator 1 Beta (*PPARGC1B*), platelet-derived growth factor receptor alpha (*PDGFRA*), Asparagine synthetase (*ASNS*), discoidin domain receptor tyrosine kinase 2 (*DDR2*), and *FGF10* were randomly selected for qRT-PCR analysis. The findings of the qRT-PCR were in line with the results of RNA sequencing results (Figure 10). The expression levels of *PDCD1*, *AREG*, *C5AR2*, *PPARGC1B* were down regulated (p<0.05) with the enrichment of *FGF10* gene. Moreover, the expression levels of *PDGFRA*, *ASNS*, *DDR2*, and *FGF10* were up-regulated with the enrichment of *FGF10* gene in adipocytes.

## DISCUSSION

Adipogenesis, being a complex biochemical process, involves differentiation of preadipocytes followed by proliferation and maturation of adipocytes. Preadipocytes, which originate from the existent group of adipocytes undergoes the process of development in response to suitable stimuli [14]. Therefore, it is essential to better understand the molecular basis of adipogenesis. Previously, we identified polymorphism of *FGF10* gene promoter and found its association with meat quality characteristics in Qinchuan beef cattle [15]. Currently, the mRNA expression level of FGF10 gene in 12 different tissues of Qinchain beef cattle showed the highest level in omasum, followed by subcutaneous fat, muscular fat, and lung tissue. Zhang et al., 2018 found the highest expression level of *FGF10* gene in lung tissue followed by thigh and breast muscles [25]. Moreover, the expression changes of *FGF10* gene during different stages of induced differentiation exhibited an increasing trend from day 2 and reached the maximum on the 4th day of induction differentiation, which then decreased on the 6th and 8th days. The results indicated that *FGF10* may play a role in the middle stage of bovine adipocyte differentiation. A similar trend was found in the mRNA expression of *FGF10* in 3T3L1 cells. The expression level increased from day 0 to day 2, and then decreased gradually until day 6 [26]. The Matsubara et al [27] reported rapid decrease in expression level of *FGF10* gene in early stage of chicken adipocyte differentiation. This variation in the expression of *FGF10* gene during adipocyte differentiation shows its function in adipocyte differentiation. Beef quality is mainly affected by fat deposition, which is closely related to adipocyte differentiation [28]. The FGFs are signaling proteins with diverse functions, especially regulate adipogenesis [29]. To further validate the role of *FGF10* on the differentiation of preadipocytes, overexpression and interference of *FGF10* were transfected into preadipocytes, and the adipocyte differentiation marker genes *CEBPα*, *PPAR*γ, *FABP4*, *FAS* and *LPL* were detected at the mRNA level. The overexpression of *FGF10* gene, down regulated the expression of *CEBPα*, *PPAR*γ, *FABP4*, and *LPL* at day 4 after induced differentiation. After overexpressing the *FGF10* gene in bovine adipocytes, the protein expression of PPARγ and FABP4 decreased significantly compared with the control group at 4th day of induced differentiation, while down regulation of FGF10 increased the expression of PPARγ and FABP4 proteins significantly at 6 days of differentiation. Based on the results of mRNA and protein level expressions, *FGF10* inhibited the expression of adipocyte differentiation marker genes. However, Previously, overexpression of *FGF10* gene in goat subcutaneous preadipocytes, enriched the expression of adipocyte differentiation marker genes such as *C/EBPα*, *LPL*, *ACACA*, *FGFR1*, *FGFR3*, *FASN*, and *ATGL* [30]. The probable reason for this variation could be due to different cell lines or different species.

To further confirm the effect of *FGF10* on the differentiation of adipocytes, after the overexpression and interference of *FGF10* gene, the oil red O staining method was used to observe and compare the morphology, and the content of triglyceride was determined. On the 4th day, oil red O staining showed that overexpression of *FGF10* produced smaller lipid droplets than that of the control group. However, lipid droplets in the FGF10-interfering treatment group were larger than those in the control group on the 6th day of induction of differentiation. The triglyceride content was also reduced in the adipocytes infected with Ad-OE-FGF10, while the triglyceride content of the interference FGF10 treatment adipocytes were relatively increased compared with the control group. This further proves that *FGF10* can inhibit triglyceride accumulation in bovine adipocytes. Therefore, we can speculate that the *FGF10* gene is a negative regulator of bovine adipocytes differentiation.

To further validate the roles of *FGF10* gene in adipocytes differentiation, deep RNA sequencing was performed, which provides a modern insight for functional genomics. A total of 1,774 DEGs were detected, including perilipin (*PLIN1*), acyl-CoAsynthetase long chain family member1 (*ACSL1*), *FABP4*, *PPARGC1B* in adipocytes infected with Ad-FGF10 or Ad-NC. The GO function analysis gives the GO function classification annotation of the gene, and it also gives the GO function significance enrichment of the gene. The GO enrichment is mainly divided into three levels of functions, namely molecular function (molecular function, GO-MF), cellular components (cellular component, GO-CC), and biological process (biological process, GO-BP). Pathway significant enrichment explores the most important biochemical metabolic pathways and signal transduction pathways involved in differential genes. In organisms, different genes coordinate with each other to exercise their biological function. Pathway-based analysis helps to further understand the interaction of genes. The results of GO enrichment analysis and KEGG pathway analysis showed that differential genes were involved in the regulation of a wide range of biological processes (such as metabolism, regulation of biological processes, and cell proliferation), and were enriched in the PPAR signaling pathway related to lipid differentiation, adipocyte regulation, fatty acid degradation and other pathways. These results indicated that *FGF10* gene plays a certain regulatory role in adipocyte differentiation. The *PPARGC1B* is a transcriptional co-stimulator of nuclear receptor *PPAR*γ, which has multiple nuclear hormone receptor binding sites. *PPARGC1B* plays a very important role in biochemical pathways such as mitochondrial proliferation and respiration, adipogenesis and adipocyte differentiation, and hepatic gluconeogenesis [31]. To function, PPARGC1B must first interact with DNA-binding transcription factors and then act on downstream targets. Many protein domains of *PPARGC1B* are used to interact with transcription factors. The *PPARGCIB* is relatively inactive, and it can affect transcription only when it combines with *PPARγ* or nuclear respiratory factor 1 (*NRF-1*) [32]. The *ACSL1* is a member of the ACSLs family, and is a key regulator of adipogenesis [33]. FABP4 promotes adipocyte differentiation and reduce lipolysis [34]. The *PLIN1* mobilize lipids in adipose tissue and is a key regulator of lipolysis and lipid storage in adipocyte [35]. Furthermore, the present study found that after overexpressing the *FGF10* gene, the expression levels of the above lipid differentiation-related genes were all down-regulated during the process of adipocyte differentiation. Interestingly, overexpression of *FGF10* gene down-regulated the transcription of FGF receptors such as *FGFRL1*, *FGFR2*, *FGFR4*, and *FGFR3* in differentiated bovine adipocytes. Previous studies show that FGFs have specific binding affinity with tyrosine kinase receptors known as FGF receptors (1–4). FGFs binding with FGFRs causes receptors dimerization and tyrosine phosphorylation, leading to activation of various signaling pathways [36], especially in adipogenesis [37]. FGF10 regulates adipogenesis through FGF receptors in various mammalian species [38,39]. Therefore, based on the findings of the current study, we can conclude that *FGF10* gene is an important negative regulator of adipogenesis and provides a foundation for the improvement of beef cattle molecular breeding program.

## Figures and Tables

**Table 1:** Comparative analysis of reference genomes

Sample	Total	Unmapped (%)	Unique mapped (%)	Multiple mapped (%)	Total mapped (%)
FG_D4-1	45,832,240	7.43	90.27	2.30	92.57
FG_D4-2	50,301,870	7.48	90.30	2.23	92.52
FG_D4-3	60,085,932	7.06	90.76	2.18	92.94
NC_D4_1	45,176,872	6.16	91.84	2.01	93.84
NC_D4_2	46,538,406	6.29	91.70	2.00	93.71
NC_D4_3	48,693,182	6.17	91.83	2.00	93.83

FG, fibroblast growth; NC, negative control.

**Table 2:** Differential transcriptomics analysis of FGF receptors in differentiated bovine adipocytes infected with Ad-FGF10 or Ad-NC

Gene	NC	FGF10	log2(fc)	Description
*FGFRL1*	6.26	1.663	−1.912085342	Fibroblast growth factor receptor like 1
*FGFR2*	5.08	2.533	−1.003791579	Fibroblast growth factor receptor 2
*FGFR1*	24.68	26.5	0.102649965	Fibroblast growth factor receptor 1
*FGFR4*	0.033	0.001	−5.058893689	Fibroblast growth factor receptor 4
*FGFR3*	0.08	0.043	−0.884522783	Fibroblast growth factor receptor 3

FGF, fibroblast growth factor; NC, negative control.

## Data Availability

The data will be available on request to the corresponding author.

## SUPPLEMENTARY MATERIAL

**Supplementary Table S1**. The sequence of bovine siFGF10 and negative control siRNA
