# Supplementary material for: RNA-Seq explores the functional role of the fibroblast growth factor 10 gene in bovine adipocytes differentiation
Source: Anim Biosci. 2023 Nov 1;37(5):929–43. doi: 10.5713/ab.23.0185 (PMC11065710; doi:10.5713/ab.23.0185)
Supplement: Supplementary file 1 [file ab-23-0185-Supplementary-Table-1.pdf]

Supplementary Table S1. The sequence of bovine siFGF10 and negative control siRNA

Si name	Sequence
Si1 FGF10-bos-142	5'-3' GCCACCAACUCCUCUUCUUTT<br>5'-3' AAGAAGAGGAGUUGGUGGCTT
Si2 FGF10-bos-348	5'-3' GGAGAUAACUUCAGUGGAATT<br>5'-3' UUCCACUGAAGUUAUCUCCTT
Si3 FGF10-bos-622	5'-3' CCGAUGGUGGUACACUCAUTT<br>5'-3' AUGAGUGUACCACCAUCGGTT
NC	5'-3' UUCUCCGAACGUGUCACGUTT<br>5'-3' ACGUGACACGUUCGGAGAATT
